# On the Dependence of the Critical Success Index (CSI) on Prevalence

**DOI:** 10.3390/diagnostics14050545

**Published:** 2024-03-05

**Authors:** Gashirai K. Mbizvo, Andrew J. Larner

**Affiliations:** 1 Pharmacology and Therapeutics, Institute of Systems, Molecular and Integrative Biology, University of Liverpool, Biosciences Building, Crown Street, Liverpool L69 7BE, UK; 2 Liverpool Centre for Cardiovascular Science, University of Liverpool and Liverpool Heart & Chest Hospital, Liverpool L14 3PE, UK; 3 Cognitive Function Clinic, The Walton Centre NHS Foundation Trust, Liverpool L9 7LJ, UK; ajlarner241@aol.com

**Keywords:** Bayes formula, binary classification, critical success index, F measure, prevalence

## Abstract

The critical success index (CSI) is an established metric used in meteorology to verify the accuracy of weather forecasts. It is defined as the ratio of hits to the sum of hits, false alarms, and misses. Translationally, CSI has gained popularity as a unitary outcome measure in various clinical situations where large numbers of true negatives may influence the interpretation of other, more traditional, outcome measures, such as specificity (Spec) and negative predictive value (NPV), or when unified interpretation of positive predictive value (PPV) and sensitivity (Sens) is needed. The derivation of CSI from measures including PPV has prompted questions as to whether and how CSI values may vary with disease prevalence (P), just as PPV estimates are dependent on P, and hence whether CSI values are generalizable between studies with differing prevalences. As no detailed study of the relation of CSI to prevalence has been undertaken hitherto, the dataset of a previously published test accuracy study of a cognitive screening instrument was interrogated to address this question. Three different methods were used to examine the change in CSI across a range of prevalences, using both the Bayes formula and equations directly relating CSI to Sens, PPV, P, and the test threshold (Q). These approaches showed that, as expected, CSI does vary with prevalence, but the dependence differs according to the method of calculation that is adopted. Bayesian rescaling of both Sens and PPV generates a concave curve, suggesting that CSI will be maximal at a particular prevalence, which may vary according to the particular dataset.

## 1. Introduction

The context of this paper is that many measures may be derived from the data cells in a 2 × 2 contingency table, which is used as the basis for evaluating any binary classification such as the outcome of a screening or diagnostic test accuracy study or a case-ascertainment algorithm [1]. Choosing the optimal measure(s) to describe the outcomes of a study may be dependent upon the nature of the available dataset.

For datasets with very large numbers of true negative (TN) outcomes in the base data, as seen for an example using routine epilepsy data [2,3,4,5,6,7,8,9,10,11,12,13,14,15,16,17,18,19,20,21,22,23,24,25,26,27,28,29,30,31], indices such as specificity (Spec), negative predictive value (NPV), and overall classification accuracy (Acc), which all feature TN values in both numerator and denominator, may be very high, indeed approaching values of 1. This is because the numbers of TN may approach the total number of observations (N), and hence swamp the values of the other cells of the 2 × 2 contingency table, namely, true positive (TP), false positive (FP), and false negative (FN).

This circumstance makes it difficult to rank the diagnostic accuracy of the corresponding case-ascertainment algorithms based on Spec, NPV, or Acc, as the figures are all similarly high [32]. In conditions such as dementia [33], motor neuron disease [34], and epilepsy [35,36], systematic reviews of the diagnostic accuracy of routine data indicate that the original studies published have largely measured the positive predictive value (PPV) and sensitivity (Sens) without measuring Spec or NPV. This is because finding true negative cases in the community to verify an absent diagnostic code in a routine dataset is a challenge for researchers, who often only have permission to study populations that have been positively coded with the disease in question. Making a judgment on the optimal case-ascertainment algorithm for a particular condition based on either PPV or Sens is challenging because PPV and Sens tend to have an inverse relationship [37], so it is difficult to know which measure to prioritize to best indicate accuracy.

There are other examples in clinical medicine where large numbers of TN may complicate the interpretation of more traditional measures such as PPV and Sens, including National Institute for Clinical Excellence criteria for 2-week-wait suspected brain and CNS cancer referrals [38], polygenic hazard scores [39], and the evaluation of cognitive screening instruments [40]. Accordingly, as we have previously indicated, a metric is needed which eschews TN and combines PPV and Sens.

As we are not aware of such a metric currently in common use in medicine, we have proposed the use of the critical success index (CSI) for this purpose. This measure, which has been intermittently reinvented over the last century, has been variously known as the ratio of verification in the context of forecasting tornadoes in meteorology [41,42,43,44,45,46,47,48,49,50], and subsequently as the Jaccard index or similarity coefficient (J) [51], the threat score [52], the Tanimoto index [53], CSI [53,54], and most recently as F* [55].

In signal detection theory, CSI is defined as the ratio of hits to the sum of hits, false alarms, and misses [40,56]. In terms of the base data of the 2 × 2 contingency table [1]:

CSI = TP/(TP + FP + FN)

= TP/(N − TN)

CSI may also be expressed in terms of PPV and Sens [1]:

CSI = 1/[(1/PPV) + (1/Sens) − 1](1)

We have demonstrated the advantages of using CSI to complement traditional diagnostic accuracy measures using real-word data in several conditions [32,40,57].

It should be noted that CSI differs from, but is related to, another measure sometimes used for similar purposes of data analysis, which is variously called the Dice coefficient, the Sørensen-Dice coefficient, or the F measure [58,59,60,61,62], defined as:

F = 2.TP/(2.TP + FP + FN)

= 2/[1/Sens + 1/PPV]

There is a monotonic relationship between CSI and F [63], such that:

F = 2CSI/(1 + CSI)

A question often raised about CSI concerns how its values relate to prevalence, P, the probability of a positive diagnosis. As we are not aware of any previous examination of this question, it merits further investigation. It is well-known that values of PPV vary with P, and thus are sensitive to class imbalance and may therefore not be generalizable between studies [63]. Since, as shown in Equation (1), CSI may be expressed in terms of PPV, a similar expectation will hold for CSI. Likewise, following from Equation (1), it may be asked whether CSI values track predominantly with Sens or PPV and whether this changes with P.

Here, we initially address two possible methods to illustrate the dependence of CSI on P, as previously suggested [57]: firstly, using the Bayes formula to recalculate PPV and then to recalculate CSI (hence, a two-step method); and secondly, using equations in which CSI is expressed directly in terms of Sens, PPV, P, and the test threshold or probability of a positive test, denoted as Q. In addition, we introduce a third method in which Sens is also rescaled by using the Bayes formula to recalculate NPV and hence Sens. This then allows CSI values to be recalculated using both rescaled PPV and Sens.

## 2. Materials and Methods

### 2.1. Dataset

The public dataset from a published screening test accuracy study of a cognitive screening instrument [64], the Mini-Addenbrooke’s Cognitive Examination (MACE) [65], was examined. In this study, MACE was administered to consecutive patient referrals (N = 755) to a dedicated cognitive disorders clinic located in a secondary care neuroscience center over the period of June 2014−December 2018 (inclusive). Other than those with a pre-existing diagnosis of dementia, there were no exclusion criteria. The diagnosis of dementia or mild cognitive impairment was made by the judgement of an experienced clinician using standard diagnostic criteria (DSM-IV; Petersen); in those without evidence of cognitive impairment, a diagnosis of subjective memory complaint (SMC) was made. MACE scores were not used to make criterion diagnoses in order to avoid review bias. Subjects gave informed consent, and the study was approved by the institute’s committee on human research (Walton Centre for Neurology and Neurosurgery Approval: N 310).

In this cohort, 114 patients received a final criterial diagnosis (DSM-IV) of dementia (P = 0.151) [65]. The original analysis of the dataset established the optimal MACE cut-off for the diagnosis of dementia to be ≤20/30 (calculated from the maximal value for the Youden index), where TP = 104, FP = 188, FN = 10, and TN = 453. Hence, at this cut-off, Sens = 0.912, Spec = 0.707, PPV = 0.356, and NPV = 0.978.

From these base data, values of CSI across a range of P values (0.1 to 0.9, in 0.1 increments) were calculated using three different methods. As CSI is dependent on PPV and Sens (Equation (1)), it is appropriate to examine how its value changes with different methods of analysis, specifically how CSI changes with the change in PPV (Method 1), with the change in Sens (Method 2), and with the changes in both PPV and Sens (Method 3).

### 2.2. Method 1: CSI Recalculated via Bayes Formula for PPV

As Sens and Spec are relatively impervious to changes, in P, being strictly columnar ratios in the 2 × 2 contingency table, PPV may be recalculated for different values of P using the Bayes formula:

PPV = Sens.P/(Sens.P) + [(1 − Spec).P′](2)

where P′ = (1 − P). Using the base data (Sens = 0.912, Spec = 0.707), the values of PPV were calculated for P values ranging from 0.1 to 0.9.

The second step in this method used the recalculated PPV values at different prevalences to recalculate CSI values according to the relation to PPV and Sens (Equation (1)).

Thus, this approach requires the sequential application of Equations (1) and (2) to the base data. Results are displayed in a table and graphically.

### 2.3. Method 2: CSI Recalculated via Its Relation to Sens, PPV, P, and Q

The dependence of CSI on P, and the probability of a positive diagnosis, may be directly expressed in terms of Sens, PPV, P, and test threshold, the probability of a positive test (Q) [1]:

CSI = 1/[(P + Q)/Sens.P] − 1(3)

CSI = 1/[(P + Q)/PPV.Q] − 1(4)

Hence, the dependence of CSI on P may be addressed by calculating its value for different values of P at chosen values of Q. Q ranges from 0−1, where Q = 0 equates to a test threshold at which there are no positives (neither TP nor FP), and Q = 1 equates to a threshold at which there are no negatives (neither TN nor FN). When Q = 0.5 in a balanced data set (P = 0.5), there are equal numbers of false positives and false negatives.

Using the base data (Sens = 0.912, PPV = 0.356), values of CSI were calculated for P values ranging from 0.1 to 0.9 to illustrate the dependence of CSI on P. Three conditions were examined: Q = 0.1 (very few false positives); Q = 0.5 (equal numbers of false positives and false negatives, if the dataset was balanced); and Q = 0.9 (very few false negatives).

Hence, this approach requires the application of either Equation (3) or Equation (4) to the base data. The results are displayed in tables and graphically.

### 2.4. Method 3: CSI Recalculated via Both Rescaled PPV and Sens

There is also a method to recalculate CSI using not only rescaled PPV, as in Method 1, but also rescaled Sens.

The Bayes formula may be used to calculate different values of NPV across the range of P values:

NPV = Spec.P′/(Spec.P′) + [(1 − Sens).P](5)

This allows for the recalculation of Sens at different P values using the equivalence shown by Kraemer, such that [66]:

(Sens − Q)/Q′ = (NPV − P′)/P

Rearranging this, values for Sens at a fixed Q may be calculated at variable P [1]:

Sens = [Q′.(NPV − P′)/P] + Q(6)

Hence, this approach requires the application of Equations (5) and (6) to the base data (Spec = 0.707; Q = 0.387 at optimal MACE cut-off of ≤20/30) to recalculate NPV and Sens, respectively.

With the rescaled Sens and the previously rescaled PPV (Table 1), it is then possible to recalculate CSI (Equation (1)). The results are displayed in a table and graphically.

## 3. Results

### 3.1. Method 1: CSI Recalculated via Bayes Formula for PPV

**Figure 1:**
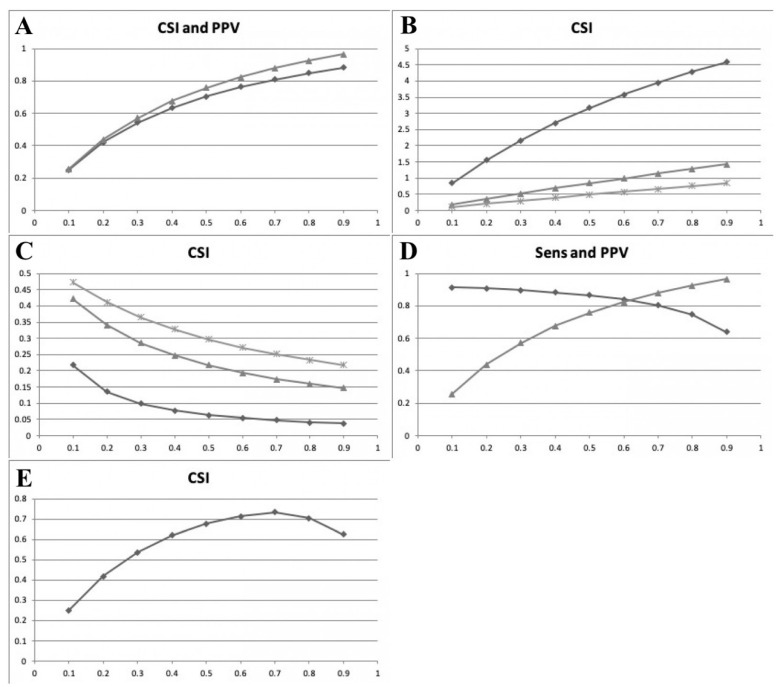
Panel of line graphs showing the study results. (**A**) Plot of CSI (♦) and PPV (▲) (y axis) for dementia diagnosis at fixed Q (Q = 0.387; MACE cut-off ≤ 20/30) versus prevalence P (x axis) calculated by sequential application of Equation (2) (Bayes formula) and Equation (1). (**B**) Plot of CSI (y axis) for dementia diagnosis at fixed Sens (0.912) and variable Q = 0.1 (♦), = 0.5 (▲), = 0.9 (*) versus prevalence P (*x* axis) calculated using Equation (3). (**C**) Plot of CSI (y axis) for dementia diagnosis at fixed PPV (0.356) and variable Q = 0.1 (♦), = 0.5 (▲), = 0.9 (*) versus prevalence P (x axis) calculated using Equation (4)**.** (**D**) Plot of Sens (♦) and PPV (▲) (*y* axis) for dementia diagnosis at fixed Q (Q = 0.387, MACE cut-off ≤ 20/30) versus prevalence P (*x* axis) calculated by application of Equations (2) and (6), respectively. (**E**) Plot of CSI (*y* axis) for dementia diagnosis at fixed Q (Q = 0.387, MACE cut-off ≤ 20/30) versus prevalence P (*x* axis), combining rescaled Sens and PPV (**D**).

Using the Bayes formula (Equation (2)), both the recalculated values of PPV and CSI increased with increasing P (Table 1; Figure 1A). This confirms the expectation evident in the Bayes formula that CSI, like PPV, is proportional to P in this formulation. This implies that the highest values of CSI will occur when P is high.

### 3.2. Method 2: CSI Recalculated via Its Relation to Sens, PPV, P, and Q

Using Equation (3) (fixed Sens value), CSI increased with increasing P (Table 2, Table 3 and Table 4, 3rd column; Figure 1B). This implies that, with a fixed Sens, the highest values of CSI will occur when P is high.

Using Equation (4) (fixed PPV value), CSI decreased with increasing P (Table 2, Table 3 and Table 4, 4th column; Figure 1C). This implies that, with a fixed PPV, the highest value of CSI will occur when P is low.

### 3.3. Method 3: CSI Recalculated via Rescaled PPV and Sens

Using this method, neither PPV nor Sens is fixed, only Q. The rescaled values (Figure 1D) show Sens decreasing with increasing P (Table 5, column 4) and PPV increasing with increasing P (Table 5, column 3; and as per Table 1 and Figure 1A).

Combining these rescaled values as per Equation (1), CSI showed a concave curve when plotted against P (Table 5 column 5, Figure 1E). CSI values approximated PPV at low values of P (as in Figure 1A), and approximated Sens values at high values of P (compare Figure 1D,E).

## 4. Discussion

This study has shown that the dependence of CSI on P differs according to the method of calculation adopted.

Using either the Bayes formula method to rescale PPV (Equation (2)) or the direct method based on Sens (Equation (3)), the CSI values increased with increasing P. In these methods, the value of Sens was fixed, but the product (Sens.P) varied with P. Hence, the CSI values increased as P increased (Figure 1A,B).

In contrast, using the direct method based on PPV (Equation (4)), the CSI values decreased as P increased. With this method, the value of PPV was fixed, and hence, the product (PPV.Q) was also fixed for each of the three chosen values of Q (Table 2, Table 3 and Table 4, 4th column). Thus, the only changing variable in this method of calculation was (P + Q), which was inversely proportional to CSI (Equation (4)). This inverse relation was also expected on the basis of the observation that test Sens and PPV changed in opposite directions with the change in test cut-off [37]. This change in opposite directions was empirically observed in the previous analysis of the dataset used in this study [64].

Using the third method, in which both PPV and Sens were rescaled via the Bayes formula, the relationship between CSI and P was shown to be a concave curve. This suggests that CSI is maximal at a particular prevalence, which may vary according to the particular dataset under examination. It was previously shown, using the same dataset, that another unitary measure based on Sens and PPV, the F measure (the harmonic mean of Sens and PPV), showed a concave curve when plotted against P, with a maximum value at P = 0.7, but falling away at both higher and lower values of P. The finding of maximal CSI at P = 0.7 in this dataset was previously predicted, since CSI and F share a monotonic relationship [1,63]. The findings suggest that, at least in this cohort, CSI values follow PPV at low values of P and follow Sens at high values of P, but this needs further investigation in other patient cohorts.

This concave relationship is simply a reflection of the fact that CSI is dependent on both P and Q, as per Equations (3) and (4). In other words, this reflects the known trade-off relationship between PPV and Sens, where one decreases as the other increases [37]. Just as paired outcome measures may be dependent on either P (PPV, NPV, and their complements) or Q (Sens, Spec, and their complements), unitary measures are also often functions of both P and Q. This is the case not only for CSI, but also for the F measure, Youden index (Y), predictive summary index (PSI), Matthews’ correlation coefficient (MCC), and the harmonic mean of Y and PSI (HMYPSI) (Table 6). All showed concave relationships to P in this dataset [1].

The major strength of this study is that it is, to our knowledge, the first to address the dependence of CSI on prevalence. A limitation is that it is based on a dataset of a single diagnostic test accuracy study. Future studies may examine other larger datasets, including those from different sources, such as case-ascertainment algorithms.

Hence, in conclusion, we suggest that there is no simple answer to the question of how CSI is dependent on P, other than that it is, and this depends on the method of calculation chosen to examine the relationship. In real-world situations, the dependence of CSI on P is not, and cannot be, independent of Q. Thus, conclusions based on outcome values of CSI (and indeed F measure) may be dataset-specific and not easily translated or generalized to other situations, as is recognized to necessarily be the case for PPV. Moreover, pragmatically, this is also the case for Sens since, although it is algebraically unrelated to P as a strictly columnar ratio in the 2 × 2 contingency table, it varies according to the heterogeneity of clinical populations (ditto Spec) [67], as is implied in the dependence of the Youden index on P (Table 6).

## Figures and Tables

**Table 1:** Values of PPV and CSI for dementia diagnosis at a fixed value of Q (MACE cut-off of ≤20/30) at various prevalence levels.

		MACE Cut-off ≤ 20/30 Sens = 0.912	
P	P′	Recalculated PPV (from Equation (2))	Recalculated CSI (from Equation (1))
0.1	0.9	0.257	0.251
0.2	0.8	0.437	0.420
0.3	0.7	0.571	0.542
0.4	0.6	0.675	0.634
0.5	0.5	0.757	0.705
0.6	0.4	0.824	0.763
0.7	0.3	0.879	0.810
0.8	0.2	0.926	0.850
0.9	0.1	0.966	0.883

**Table 2:** Values of CSI for dementia diagnosis at fixed values of Q = 0.1 and either Sens (0.912) or PPV (0.356) at various prevalence levels.

P	P + Q	CSI (Equation (3)) Sens = 0.912	CSI (Equation (4)) PPV = 0.356
0.1	0.2	0.838	0.217
0.2	0.3	1.55	0.135
0.3	0.4	2.16	0.098
0.4	0.5	2.70	0.077
0.5	0.6	3.17	0.063
0.6	0.7	3.58	0.054
0.7	0.8	3.95	0.047
0.8	0.9	4.28	0.041
0.9	1.0	4.58	0.037

**Table 3:** Values of CSI for dementia diagnosis at fixed values of Q = 0.5 and either Sens (0.912) or PPV (0.356) at various prevalence levels.

P	P + Q	CSI (Equation (3)) Sens = 0.912	CSI (Equation (4)) PPV = 0.356
0.1	0.6	0.179	0.421
0.2	0.7	0.352	0.341
0.3	0.8	0.520	0.286
0.4	0.9	0.682	0.247
0.5	1.0	0.838	0.217
0.6	1.1	0.990	0.193
0.7	1.2	1.14	0.174
0.8	1.3	1.28	0.159
0.9	1.4	1.42	0.146

**Table 4:** Values of CSI for dementia diagnosis at fixed values of Q = 0.9 and either Sens (0.912) or PPV (0.356) at various prevalence levels.

P	P + Q	CSI (Equation (3)) Sens = 0.912	CSI (Equation (4)) PPV = 0.356
0.1	1.0	0.100	0.473
0.2	1.1	0.199	0.412
0.3	1.2	0.295	0.365
0.4	1.3	0.390	0.328
0.5	1.4	0.483	0.297
0.6	1.5	0.574	0.272
0.7	1.6	0.664	0.251
0.8	1.7	0.752	0.233
0.9	1.8	0.838	0.217

**Table 5:** Values of recalculated PPV (as per Table 1), Sens, and CSI for dementia diagnosis at various prevalence levels.

P	P′	Recalculated PPV (from Equation (2))	Recalculated Sens (from Equation (6))	Recalculated CSI (from Equation (1))
0.1	0.9	0.257	0.914	0.251
0.2	0.8	0.437	0.908	0.418
0.3	0.7	0.571	0.896	0.536
0.4	0.6	0.675	0.884	0.620
0.5	0.5	0.757	0.865	0.677
0.6	0.4	0.824	0.840	0.712
0.7	0.3	0.879	0.803	0.723
0.8	0.2	0.926	0.746	0.704
0.9	0.1	0.966	0.640	0.625

**Table 6:** Summary of dependence of unitary measures on P and Q.

Unitary Measure	Dependence on P and Q
Critical success index (CSI)	CSI = 1/[(P + Q)/Sens.P] − 1 CSI = 1/[(P + Q)/PPV.Q] − 1
F measure (F)	F = 2.Sens.P/(Q + P) F = 2.PPV.Q/(Q + P)
Youden index (Y)	Y = (Sens − Q)/P′ Y = (Spec − Q′)/P Y = (Q − Q^2^/P − P^2^).PSI
Predictive summary index (PSI)	PSI = (PPV − P)/Q′ PSI = (NPV − P′)/Q PSI = (P − P^2^/Q − Q^2^).Y
Matthews’ correlation coefficient (MCC)	MCC = √(P − P^2^/Q − Q^2^).Y MCC = √(Q − Q^2^/P − P^2^).PSI
Harmonic mean of Y and PSI (HMYPSI)	HMYPSI = 2/(1/Y).[(1 + (Q − Q^2^)/(P − P^2^)] HMYPSI= 2/(1/PSI).[(P − P^2^)/(Q − Q^2^) + 1]

## Data Availability

Anonymized base data in this secondary analysis are available for public use from the authors of the original study on a CCBY basis [64].
